# Fly in the Ointment: Host-Specificity Challenges for *Botanophila turcica,* a Candidate Agent for the Biological Control of Saffron Thistle in Australia

**DOI:** 10.3390/insects16040357

**Published:** 2025-03-28

**Authors:** Vincent Lesieur, Thierry Thomann, Mireille Jourdan, Javid Kashefi, Marie-Claude Bon

**Affiliations:** 1 CSIRO European Laboratory, 830 Avenue du Campus Agropolis, 34980 Montferrier sur Lez, France; thierry.thomann@csiro.au (T.T.); mireille.jourdan@csiro.au (M.J.); 2 European Biological Control Laboratory, USDA-ARS, 810 Avenue du Campus Agropolis, 34980 Montferrier sur Lez, France; jkashefi@ars-ebcl.org (J.K.); mcbon@ars-ebcl.org (M.-C.B.)

**Keywords:** *Carthamus lanatus*, *Carthamus tinctorius*, *Centaurea solstitialis*, *Botanophila turcica*, *Botanophila brunneilinea*, host-specificity testing, weed biocontrol

## Abstract

In classical biological control of weeds, the risk posed by a candidate agent to close relatives of the target weed in the intended area of release is a key criterion (i.e., candidate agents that demonstrate a high degree of host specificity). In this study, we investigated if the rosette crown-feeding fly *Botanophila turcica* Hennig (Diptera: Anthomyiidae) could meet this criterion and thus be considered a good candidate to control saffron thistle *Carthamus lanatus* L. (Asteraceae: Cardueae) in Australia. Previous studies indicated that *B. turcica* is specific to *Ca. lanatus* and did not infest the closely related crop, safflower (*Carthamus tinctorius* L.). However, more recent field observations made in Greece reported that *B. turcica* infested safflower in cultivated fields. To determine if *B. turcica* is safe for release as a biocontrol agent, we re-examined the host range of *B. turcica* by performing new host-specificity testing combined with field surveys carried out in the south of France during two consecutive years. We also investigated the species identity of the flies by comparing DNA sequences (*COI* barcode region) of specimens collected in France from *Ca. lanatus* and *Centaurea solstitialis* L. with those from Greece collected from *Ce. solstitialis* and *Centaurea diffusa* Lam. Our *COI* analyses confirmed that French and Greek samples identified as *B. turcica* belonged to the same species, while a second group of Greek samples matched *B. brunneilinea*, indicating two distinct species. Our results also demonstrated that *B. turcica* has a wider host range than previously suggested. Laboratory testing indicated that *Ca. lanatus*, *Ca. tinctorius,* and *Ce. solstitialis* are suitable for the development of *B. turcica*. Field surveys also revealed that *Ce. diffusa* is part of the host range of the fly. Based on the results reported here, *B. turcica* may have the potential to control both the target weed, *Ca. lanatus,* and *Ce. Solstitialis,* but it may also be a threat to safflower, *Ca. tinctorius*. Further investigations to assess under what conditions *B. turcica* attacks *Ca. tinctorius* may help clarify the level of risk to Australian growers.

## Simple Summary

This study examines the safety of using the fly *Botanophila turcica* to control the weed saffron thistle (*Carthamus lanatus*) in Australia. While previous research suggested the fly only targeted saffron thistle, new observations in Greece showed it also infested safflower (*Carthamus tinctorius*) crops. To verify this, we conducted new tests and field surveys in France and analysed DNA from flies collected in France and Greece. Our findings revealed that *B. turcica* can develop on saffron thistle, safflower, and other related plants. This means that while *B. turcica* could help control saffron thistle, it also poses a risk to safflower crops. Further research is needed to understand the conditions under which *B. turcica* attacks safflower to assess the potential risk to Australian safflower growers.

## 1. Introduction

Classical biological control (CBC) of invasive alien plants (IAPs), which consists of the deliberate introduction of a biological control agent (BCA) in the invaded range of the IAP, is considered a sustainable, self-perpetuating, and effective management method of IAPs [1,2]. According to Hinz et al. [3], approximately 25% of CBC programmes of IAPs have achieved complete control, and 50–70% of CBC programmes have achieved at least substantial control. Several studies also suggest that CBC programmes can produce significant economic returns. For example, CBC programs of IAPs in Australia have returned an estimated AUD 23 in benefits for every dollar invested in their development [4]. However, while CBC of IAPs may have favourable long-term social and economic outcomes, a CBC program is a succession of several steps that can be time- and resource-consuming [5]. Consequently, many studies focused on the selection of BCAs and identified several criteria for considering when the candidate is a “good candidate” that justifies further investigations [5,6,7,8,9,10]. Among them, (1) the potential to be damaging (i.e., candidate agents that lower the reproductive potential of the host plant) and (2) the safety (i.e., candidate agents that demonstrate a high degree of host specificity) are considered key criteria. Thus, it is important to produce data to determine whether the candidate agent meets the key criteria. In this study, we investigated whether the rosette crown-feeding fly *Botanophila turcica* Hennig (Diptera: Anthomyiidae) could meet these criteria and thus be considered a good candidate to control the saffron thistle *Carthamus lanatus* L. (Asteraceae: Cardueae) in Australia.

Native to southern Europe, *Ca. lanatus* is the most prevalent thistle in the southern regions of Australia, occurring in a wide range of climates and habitats, including pastures and cropping areas, especially cereals [11]. In the early 2000s, it was estimated that the yearly cost of *Ca. lanatus* to the New South Wales (NSW) meat and wool industries exceeded AUD 110 million [11]. This annual weed reproduces by large seeds, which are well adapted to persist for several years in the soil in Australia, and does not require a cold period to trigger sprouting [12], making the populations difficult to manage. Conventional methods involving herbicides or pasture replacement have been judged not feasible given the vast areas of extensive grazing land infested [11]. Strategic grazing (i.e., rest periods combined with periods of grazing at stocking rates that encourage sheep to eat it) can help control *Ca. lanatus,* but this method is better adapted to small paddocks [13]. It has been suggested that *Ca. lanatus* infestations will remain beyond control or containment unless CBC is implemented [14].

Previous surveys, conducted in the Mediterranean regions of Europe in the early 1990s for CBC of the weed, found four promising BCAs, and *B. turcica* was considered the most promising [15]. Larvae of *B. turcica* feed in the crown of the rosettes of immature plants. The fly is a univoltine species that oviposits eggs individually close to the collar of young plants from mid-October to mid-March [16]. First-instar larvae burrow into and feed on the rosette meristem and root crown, where they develop through three successive larval instars. It has been shown that crown feeders are effective control agents relative to other BCA guilds [2]. Field surveys performed in Southern France showed that *B. turcica* reduced seed production of *Ca. lanatus* [15,17], meeting the first criterion of being a promising BCA (i.e., potential to be damaging).

Regarding the second criterion of being safe, previous field surveys and host-specificity testing suggested that *B. turcica* has a limited host range. In southern France, *B. turcica* had never been observed attacking cultivated safflower, *Carthamus tinctorius* L. [15], an annual plant closely related to the weed. Host-specificity testing (no-choice larval feeding tests) in France also showed that *B. turcica* was not able to complete its full development on *Ca. tinctorius* [16]. Likewise, under choice conditions, no oviposition by *B. turcica* females was observed on *Ca. tinctorius* [16]. However, Tsialtas et al. [18] reported the first infestations of *B. turcica* on *Ca. tinctorius* in central and northern Greece. While *Ca. tinctorius* is not widely grown in Australia at this time [19], it is unlikely that off-target damage on this crop from a BCA would be acceptable to Australian regulators. Furthermore, two *Botanophila* species have been reared in Greece from larvae collected feeding on the centre of rosettes of the yellow starthistle, *Centaurea solstitialis* L., and diffuse knapweed, *Centaurea diffusa* Lam. (Asteraceae: Cardueae) (unpublished results). One of these fly species was presumed to be *B. turcica* based on DNA-based identification (unpublished results). To our knowledge, these plant species belonging to the same subtribe as *Ca. lanatus* (i.e., Centaureinae) were not reported as host plants for the fly before, suggesting that the host range is wider than previously thought.

The goal of this study is a step in determining if *B. turcica* meets the second criterion as follows: to be safe for release as a candidate agent to control *Ca. lanatus* in Australia. Specifically, we re-examined the host range of *B. turcica* by performing new host-specificity testing combined with field surveys carried out in the south of France during two consecutive years. We also clarified the identity of French and Greek fly specimens by comparing DNA sequences (*COI* barcode region) of specimens from Greece collected from *Ce. solstitialis* L. and *Ce. diffusa* with those of specimens collected in France in *Ca. lanatus* and *Ce. solstitialis* to determine if those specimens belong to the same or to a different species.

## 2. Materials and Methods

### 2.1. Molecular Characterization

Ethanol-preserved adults (*n* = 4) or eggs (*n* = 2) and larvae (*n* = 27) were used in this study. All information about the specimens is reported in Table 1. The French specimens consisted of individuals collected in the field around Montpellier (i.e., the three sites detailed in the Field surveys section) on both *Ca. lanatus* and *Ce. solstitialis*. We included in the molecular analysis one individual that emerged from our no-choice tests and that completed its larval development in *Ca. tinctorius* to confirm that the specimen was indeed the same genetic entity, *B. turcica*.

We also included a voucher specimen of *B. turcica* collected in 1995 (larva collected on *Ca. lanatus* and adult emerged in May) at Murles (France) and formally identified by an expert taxonomist, V. Michelsen (Natural History Museum of Denmark, Copenhagen, Denmark).

Specimens from Greece were collected as eggs or larvae at two locations on *Ce. solstitialis* and *Ce. diffusa* and preserved in ethanol at −24 °C until further use (Table 1).

For all specimens, extraction of total genomic DNA was performed using Qiagen DNeasy Blood & Tissue kit following the manufacturer’s protocol.

For specimens collected in France, PCR amplifications of the standard DNA barcoding region of cytochrome oxidase I (*COI*) were performed using universal primers LCO1490/HCO2198 [20]. For specimens collected in Greece, primer sets TW-J-1301/C1-N-2353 [21] and C1-J-2183/TL2-N-3014 [22] were used to amplify two *COI* segments, which were assembled into a full-length *COI.* The PCR products were purified and sequenced in both directions using the same sets of PCR primers by Genoscreen, France (https://www.genoscreen.fr/, accessed on 15 January 2025). Consensus sequences were generated from forward and reverse DNA sequence reads, manually edited with Chromas Lite (Technelysium Pty Ltd., South Brisbane, Australia) and compared with those in GenBank using the BLAST algorithm (https://www.ncbi.nlm.nih.gov/genbank/, accessed on 15 January 2025) and the Barcode Of Life Database (BOLD) using the BOLD identification engine (http://www.boldsystems.org/index.php, accessed on 15 January 2025). As BLAST and BOLD hit scores evidenced a high similarity between some of our sequences and *Botanophila brunneilinea* Zetterstedt, we added two *B. brunneilinea* sequences to our study dataset of 33 sequences (BOLD identifier: BIOUG53712-E12 and GenBank accession number: MZ622770). Three *COI* sequences of three *Botanophila* species (*B. biciliaris*, BOLD identifier: BIOUG05264-C05; *B. fugax*, GenBank accession number: MZ609435; and *B. helviana*, BOLD identifier: CBG-A11493-D03) were included as outgroups in the phylogenetic analysis.

Phylogenetic relationships among sequences were estimated with (1) the Neighbour Joining (NJ) method and (2) a maximum-likelihood (ML) approach, both implemented in the MEGA X software [23]. The best-fit model of sequence evolution was selected using the Bayesian Information Criterion (BIC). Node support values were assessed via bootstrap resampling using 1000 replicates. The pairwise distances between genetic groups were examined with the Kimura 2-parameters distance (K2P), which is often recommended in the frame of barcoding studies.

Although the use of arbitrary genetic distance thresholds for delimiting species using *COI* is quite popular in the literature, it remains problematic in Diptera given the wide overlap observed between intra- and interspecific distances as highlighted by [24]. We estimated the value of the distance threshold for *Botanophila* using an approach that is described in the Supplementary Material (Section S1).

We also used three different methods to delimit species to confirm that the taxonomic unit considered *B. turcica* in our study corresponds to only one species and not a set of cryptic species. We used Automatic Barcode Gap Discovery (ABGD) [25], the Bayesian implementation of Poisson tree processes (PTPs) model for species delimitation [26], and the Assemble Species by Automatic Partitioning (ASAP) method [27]. More details on each method are provided in the Supplementary Material (Section S1).

### 2.2. Field Surveys

Two study sites, located at Viols-en-Laval (43°45′06.3″ N 3°43′15.4″ E) and Saint-Clément-de-Rivière (43°41′26.6″ N 3°50′47.1″ E), Hérault, France, with natural populations of *Ca. lanatus*, were selected for fortnightly visits for two consecutive years. Those sites were surveyed from early November 2020 to early April 2021 and from mid-November 2021 to early February 2022. At Viols-en-Laval, *Ca. lanatus* and its closely related species *Ce. solstitialis* are sympatric and present at high densities, while at Saint-Clément-de-Rivière, only *Ca. lanatus* was present. In the field session 2021–2022, we also added another site, located at Le Triadou (43°44′49.8″ N 3°51′30.3″ E; Hérault, France), where only *Ce. solstitialis* was present (Table 2).

After seedling emergence in autumn, the two study sites were visited every two weeks, and each time, 30 rosettes of *Ca. lanatus* were haphazardly collected in each site. Back in the laboratory, all collected plants were inspected meticulously for the presence of *B. turcica*’s eggs or larvae. A series of growth parameters (i.e., the diameter of the plant, the diameter of the collar, and the number of leaves per plant) were measured. The same method was applied for *Ce. solstitialis*.

### 2.3. Host-Specificity Testing

#### 2.3.1. Plant Material

An abridged plant test list to assess the host range of *B. turcica* was developed based on currently accepted phylogenetic information available in the literature [28,29,30] (Table 3). The species listed were selected based on their phylogenetic relationship to the target weed [31,32]. *Carthamus lanatus* belongs to the tribe Cardueae in the subfamily Carduoideae of the Asteraceae family. Of the 16 genera of the tribe Cardueae recorded for Australia, 14 are naturalized and only two are native [28]. The abridged test list was composed of the following five plant species: *Ca. lanatus*, *Ca. tinctorius* var. Orange Grenade, *Ce. solstitialis*, *Onopordum illyricum* L., and *Rhaponticum australe* (Gaudich.) Sojak—the closest Australian native species. This list did not include all the species tested by Vitou, Briese, Sheppard, and Thomann [16], but only the species for which previous results were ambiguous or incomplete (i.e., *O. illyricum*).

All plants used in this study were propagated from seeds. Seeds of *Ca. lanatus*, *Ce. solstitialis*, and *O. illyricum* were collected in southern France in early October 2020, while seeds of *Ca. tinctorius* were obtained from a commercial outlet. Seeds of the Australian native *R. australe* were sourced in Queensland (Australia) in February 2019. For germination, seeds were placed on moist filter paper in the dark at 24 °C (±1 °C). For *Ca. lanatus* and *O. illyricum*, seeds were previously scarified. Young seedlings were then transferred to free-draining pots (9 × 9 cm wide and 10 cm deep) containing standard horticultural grade compost (Neuhaus Humin Substrat N6; Klasmann-Deilmann GmbH, Geeste, Germany). Plants were then grown in an unheated glasshouse under natural light.

#### 2.3.2. No-Choice Tests

All plants used in the no-choice tests were at the rosette stage, except for *R. australe* and *Ca. tinctorius,* which do not form a true rosette. Moreover, Tsialtas, Michelsen, and Koveos [18] did not clearly report the specific stage of *Ca. tinctorius* that was infested in Greece. Consequently, in our study, three stages were tested (seedlings = 5–6 leaves, 5 cm in height; mature plants with only leaves = 25–30 cm in height; and mature plants with flower buds = 40–45 cm in height). No-choice tests consisted of ten replicates per plant species, except for *R. australe* and *Ca. tinctorius* plants with flower buds, where only seven replicates were used.

No-choice tests were performed by transferring freshly emerged larvae (1–2 days old) from eggs collected in the field (Saint-Clément-de-Rivière site; see Section 2.2 for more details). Eggs were removed from the *Ca. lanatus* rosettes on which they had been collected, transferred onto wet filter paper in Petri dishes and kept at 18 °C (±1 °C) and a 12 h photoperiod (LED lights). Petri dishes were inspected daily to check for newly hatched larvae.

One larva was transferred with a moist fine paintbrush onto the leaf axils of a plant. Before and after inoculation, the plants were sprayed with water using a hand-held sprayer to avoid larval desiccation. The plants were then placed in screened cages (80 × 50 × 80 cm) in a large building hangar under ambient conditions (temperatures ranged between 5 °C and 27 °C, with a mean of 15 °C) and natural light. Plants were sprayed every day until the end of the experiment.

Two weeks after inoculation, when damage caused by larval development is clearly visible on *Ca. lanatus* (pers. obs.), every plant was inspected for signs of larval development. If no damage was observed, plants were held for an additional two weeks. At that time, if no damage was observed, plants were dissected to confirm that there was no larval development. Plants with damage were held in a screened cage for an additional 45 days (at that time, all larvae were expected to have reached the pupal stage [16]). Plants were dissected, and the soil was sieved to look for the pupa. All pupae were kept individually in Petri dishes maintained at 18 °C (±1 °C) and a 12 h photoperiod (LED lights) until adult emergence.

#### 2.3.3. Choice Tests

Choice tests involved oviposition tests to determine the plant species preferences of female *B. turcica* for egg-laying. Larval choice feeding tests are inappropriate for this species, as larvae do not leave the plant on which oviposition occurred [16]. Only the plant species where larval development was observed in no-choice tests were included in these tests.

Tests were conducted with adult flies that emerged from eggs or larvae collected from *Ca. lanatus* in the field around Montpellier and were then transferred to new *Ca. lanatus* plants in the laboratory. One male and two presumed mated females were placed in screened cages (40 × 40 × 60 cm) containing one potted plant of the target weed (i.e., *Ca. lanatus*) and a non-target plant species (i.e., *Ca. tinctorius* or *Ce. solstitialis*) for two weeks. We also included a treatment where we exposed the two non-target plant species (*Ca. tinctorius* vs. *Ce. solstitialis*) to gain insight into the behaviour of the fly in the absence of the target species. In each cage, a mixture of honey, pollen, yeast, and vitamins was provided to the flies for food, and moist filter paper was added as a source of water.

*Carthamus tinctorius* does not form a true rosette compared to *Ca. lanatus* and *Ce. Solstitialis*; consequently, plants of *Ca. tinctorius* consisted of plants with young flower buds (the most susceptible plant stage suggested by our no-choice tests), while *Ca. lanatus* and *Ce. solstitialis* were at the rosette stage. Plants were all about the same age, and the rosettes of *Ca. lanatus* and *Ce. solstitialis* were all about the same size. In the cages, plants were positioned to not touch each other to prevent any potential influence on the behaviour of the fly. The tests were performed in a controlled temperature room at 18 °C (±1 °C) and a 12 h photoperiod (LED lights). Plants were sprayed with water every day until the end of experimentation. Any dead female was replaced. Choice tests consisted of ten replicates. Each week, plants were inspected, and eggs were counted and gently removed from the plants with a moist paintbrush to ensure that no young larvae developed; the presence of a larva could either stimulate or inhibit additional oviposition and thus introduce bias into our experiment.

### 2.4. Statistical Analyses

All analyses were performed in R 4.4.2 [33] via the RStudio interface (v. 2022.07.2).

Field surveys: The number of eggs per plant was analysed with a generalized linear model (GLM) with a Poisson distribution to test for differences between plant species. A similar analysis was carried out for the number of larvae per plant. A generalized linear mixed model (GLMM) was used to analyse the relationship between the number of eggs (response variable) and several predictor variables: rosette diameter, collar diameter, number of leaves, and the host plant species. The site was included as a random effect in the model. The analysis was performed using the lme4 1.1-31 package [34].

A subsample dataset including only data from the site “Viols-en-Laval”, where both *Ce. solstitialis* and *Ca. lanatus* co-occurred, was used to analyse the proportion of attacked plants (i.e., the proportion of plants with eggs, larvae, or both). This proportion was analysed using a GLM with a binomial distribution. Terms included in the model were host species, year, and their interaction.

All GLMs were checked for overdispersion. All GLMs were performed with the *lme4* package, and the ANOVA method was used to compute test statistics [34].

No-choice test: A Cox proportional hazards model was fitted to examine test plant species on larval survival using the package survminer 0.4.9 [35]. The proportions of plants showing signs of larval development between plant species were compared using pairwise Fisher’s exact tests with the FDR (false discovery rate) correction for multiple comparisons.

Oviposition choice tests: The number of eggs laid per female during the three weeks of the choice oviposition test was analysed using a Mann–Whitney test because of the nonnormal distributions of the data.

## 3. Results

### 3.1. Molecular Characterization

**Figure 1:**
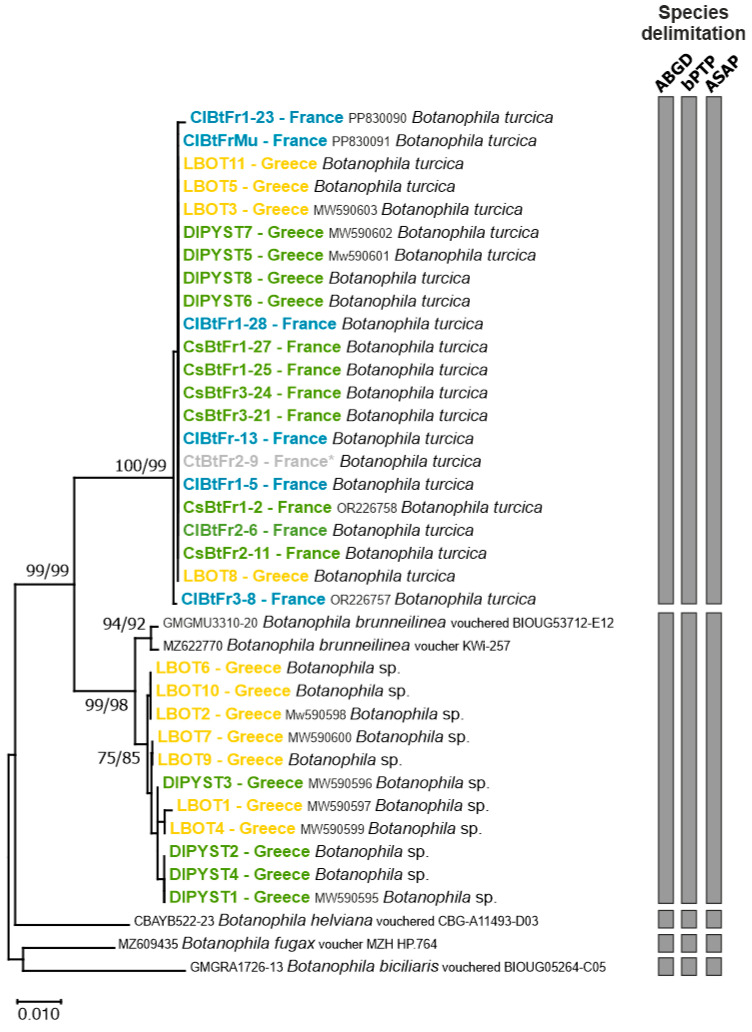
Neighbour-Joining (NJ) tree based on the *COI* sequences of *Botanophila* specimens. Significant values of bootstrap support (>75%) obtained for both NJ and ML are shown at the nodes. The scale bar represents the number of nucleotide substitutions per site. Names of terminals indicate codes of the samples. Colours represent the host plants: blue = *Carthamus lanatus*; grey = *Carthamus tinctorius* (* is for individuals that emerged from host-range testing, but the egg was originally collected on *Carthamus lanatus*); green = *Centaurea solstitialis* and orange = *Centaurea diffusa*. Grey vertical bars represent the results of the species delimitation methods (i.e., ABGD, bPTP, and ASAP procedures [25,26,27]).

The final alignment comprised 38 sequences of 628 bp. All sequences obtained in the present study were deposited in GenBank (accession numbers provided in Table 1). Although the most appropriate model of *COI* sequence evolution was the Tamura 3-parameter, we selected the K2P model for the phylogenetic reconstructions to be consistent with the pairwise distances and species delimitation methods that do not use T92. Tree topologies obtained with NJ and ML methods were congruent and revealed two well-supported genetic groups (Figure 1). The genetic divergence between these two groups was 4.32 ± 0.82%, a value expected between *Botanophila* species (Supplementary Material, Figure S1) and indicative of two different species. Moreover, the three different methods used to delimit species clearly supported the two groups as two different *Botanophila* species.

All French individuals collected on *Ca. lanatus* or *Ce. solstitialis*, including individuals used in our no-choice tests, clustered with the sequence of the adult morphologically identified by the expert taxonomist (V. Michelsen, Natural History Museum of Denmark, Copenhagen, Denmark) as *Botanophila turcica*. This genetic group also included sequences of four individuals collected in Greece on *Ce. solstitialis* and four individuals reared from *Ce. diffusa*. Those eight Greek samples had similar *COI* sequences as the most common haplotype found in France. All species delimitation methods used were congruent and clearly suggested that the samples considered *B. turcica* in our study belonged to the same species, whatever the host plant or the sampling area (Figure 1).

The second group was composed of sequences of 11 individuals exclusively collected in Greece on *Ce. solstitialis* (*n* = 4) and *C. diffusa* (*n* = 7) and closely matched the sequences of voucher specimens of *B. brunneilinea*. All species delimitation methods indicated that these individuals should be considered belonging to *B. brunneilinea.*

### 3.2. Field Surveys

A total of 1290 plants were collected during the surveys, and *B. turcica* eggs were observed in the field from mid-November until mid-February, and larvae were found throughout the survey across both years. We did not observe any significant differences between *Ca. lanatus* and *Ce. solstitialis* in terms of the seasonal dynamics of the fly (Supplementary Material, Figure S2). However, at Viols en Laval, *B. turcica* was detected on *Ca. lanatus* two weeks earlier than on *Ce. solstitialis*.

We found eggs and larvae on both *Ca. lanatus* and *Ce. solstitialis* on each sampling site (Table 2). The highest infestation rate was observed in 2021 at Saint-Clément-de-Rivière on *Ca. lanatus* (27.3%), while the lowest was found at Viols-en-Laval on *Ce. solstitialis* (2.59%, Table 2). At the Viols-en-Laval site, where the two plant species co-occurred, the infestation rate on *Ca. lanatus* was ca. two times higher than on *Ce. solstitialis* (*χ*^2^ = 4.91; *d.f.* = 1; *p* = 0.027), and we did not detect significant effects of year or the interaction between plant species and year (*χ*^2^ = 0.084; *d.f.* = 1; *p* = 0.771 and *χ*^2^ = 1.942; *d.f.* = 1; *p* = 0.163, respectively).

For both plant species, most of the rosettes with eggs were bearing only one egg (*Ca. lanatus* 1.53 ± 0.06 and *Ce. solstitialis* 1.14 ± 0.08). Only a few rosettes of *Ce. solstitialis* (*n* = 2) were found with more than one egg, while up to six eggs were observed on *Ca. lanatus* rosettes, but no statistical differences were found between plant species (*χ*^2^ = 0.30; *d.f.* = 1; *p* = 0.579). Rosette diameter was a significant predictor of egg presence (estimate = 0.137, *p* < 0.001), indicating that larger rosettes are associated with a higher likelihood of egg presence. In contrast, other plant parameters, such as collar diameter (estimate = −0.174, *p* = 0.141) and number of leaves (estimate = 0.003, *p* = 0.929), did not have a significant effect. Additionally, *Ce. solstitialis* had a significantly lower likelihood of egg presence compared to *Ca. lanatus* (estimate = −1.063, *p* = 0.027).

No rosettes were found supporting more than one larva for *Ce. Solstitialis,* and a few rosettes of *Ca. lanatus* (*n* = 6) were observed with two developing larvae, but the plant species had no significant effect on the number of larvae per rosette (*χ*^2^ = 0.07; *d.f.* = 1; *p* = 0.795).

### 3.3. Host-Specificity Testing

#### 3.3.1. No-Choice Tests

**Figure 2:**
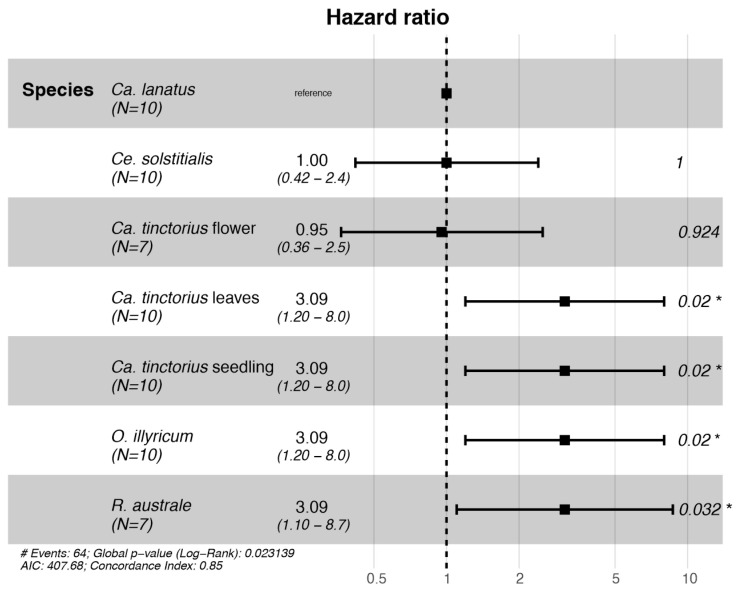
Forest plot of a Cox proportional hazards model fitted with *Botanophila turcica* larvae no-choice survival data. Hazard ratios > 1 indicate an increased risk of death to larvae, whereas hazard ratios < 1 indicate a decreased risk. * *p* < 0.05.

Larval survival was significantly affected by the plant species (Cox proportional hazards model: χ^2^ = 14.65; *d.f.* = 6; *p* = 0.023; Figure 2). Successful larval development was recorded on *Ca. lanatus* and *Ca. tinctorius* plants with flower buds and *Ce. solstitialis*. The Cox proportional hazards model found that larvae placed on *O. illyricum*, *R. australe,* and *Ca. tinctorius* (except the treatment *Ca. tinctorius* plants with flower buds) had an increased risk of dying.

The proportions of plants showing signs of larval development were also affected by the plant species (Fisher exact test, *p* < 0.001; Table 3). Typical damage caused by larval feeding was observed on all species/treatments except on *O. illyricum* and *Ca. tinctorius* bolted plants with only leaves. For *Ca. tinctorius* seedlings and *R. australe*, no larvae completed development. For *Ca. tinctorius* seedlings, the plants where larval development was initiated (30% of the tested plants) died prematurely, impeding complete larval development. Larval damage observed on *R. australe* (42% of the tested plants) was very low, corresponding to the mining of the very young larvae, and larvae died only a few days after inoculation.

#### 3.3.2. Choice Tests

**Figure 3:**
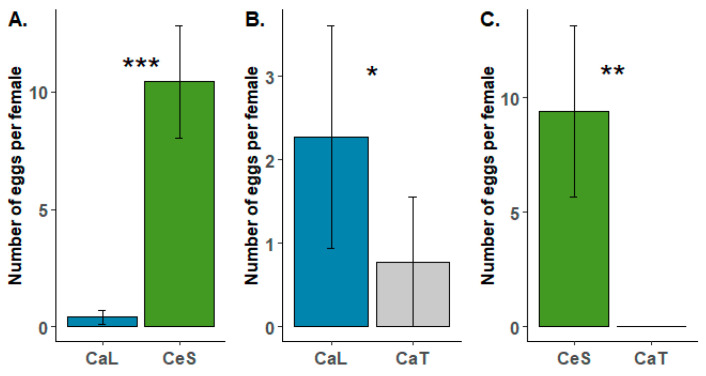
Results of the oviposition choice tests of *Botanophila turcica* females under choice conditions for two weeks. (**A**) *Carthamus lanatus* (CaL) vs. *Centaurea solstitialis* (CeS); (**B**) *Carthamus lanatus* vs. *Carthamus tinctorius* (CaT) and (**C**) *Carthamus tinctorius* vs. *Centaurea solstitialis*. Each test consisted of ten replicates. Significance levels of Mann–Whitney tests: * *p* < 0.05; ** *p* < 0.01; *** *p* < 0.001. Please note differences in scale in the *y*-axis of (**B**), relative to (**A**,**C**).

During the oviposition choice tests, females laid their eggs on all tested species (*Ca. lanatus* vs. *Ca. tinctorius*, *Ca. lanatus* vs. *Ce. solstitialis* and *Ca. tinctorius* vs. *Ce. solstitialis*). Preference for oviposition was observed for the target weed *Ca. lanatus* compared to *Ca. tinctorius* (Figure 3B). The difference observed during the tests was statistically supported (*U* = 62; *p* = 0.034), but the number of eggs laid per female during the test was quite low in both species (2.28 ± 1.34 in *Ca. lanatus* compared to 0.78 ± 0.78 in *Ca. tinctorius*). In the two other treatments where *Ce. solstitialis* was included, the number of eggs laid per female during the tests was much higher (Figure 3A,C). Testing revealed that *B. turcica* has a strong preference for *Ce. solstitialis* (Figure 3A,C). The number of eggs laid per female on *Ce. solstitialis* (10.40 ± 2.39) during the tests largely differed (*U* = 0; *p* < 0.001) compared to the one observed on *Ca. lanatus* (0.40 ± 0.28), and no oviposition occurred on *Ca. tinctorius* in the *Ca. tinctorius* vs. *Ce. solstitialis* treatment, while 9.42 eggs (± 3.76) were laid per female on *Ce. solstitialis* (Figure 3C).

## 4. Discussion

The results provided by our investigation showed that *Botanophila turcica* has a wider host range than previously suggested [16]. The no-choice test results indicated that *Ca. lanatus* and the closely related crop, *Ca. tinctorius*, are suitable for the development of *B. turcica*. These results tend to confirm more recent observations made in Greece, where *B. turcica* was found infesting plants in cultivated fields of *Ca. tinctorius* [18]. Unfortunately, we were not able to collect fresh specimens or retrieve dried specimens that emerged from *Ca. tinctorius* in Greece mentioned by Tsialtas, Michelsen, and Koveos [18], but all available information (from the literature and the present study) strongly suggests that the fly observed on *Ca. tinctorius* crops corresponded to *B. turcica*.

Choice tests shed light on the potential preference of *B. turcica* females for oviposition. As the larval stage does not leave the plant on which oviposition occurred, testing the preference of the females for oviposition between the target weed and the non-target plant species is essential to assess the risk for non-target plants [7,10]. *Botanophila turcica* females showed a significant preference for the target weed, *Ca. lanatus*, compared to the crop, *Ca. tinctorius*, suggesting a low threat to cultivated crops. In Australia, *Ca. tinctorius* has received focused attention as an industrial oilseed and potentially represents a significant new crop industry for the northern region. It was previously suggested that there is a phenological incompatibility between *Ca. tinctorius* and *B. turcica* [15]; the egg stock of the females being laid during autumn before *Ca. tinctorius* is planted in the field. Given our results and the observations made in Greece, this inference should be tempered. We found eggs on *Ca. lanatus* over more than three months in autumn and winter. The incompatibility may be true only for *Ca. tinctorius* varieties sown later (i.e., in spring). *Carthamus tinctorius* sowing time may depend on several parameters, such as the geographical range or the *Ca. tinctorius* varieties. In central and northern NSW, it is recommended to sow *Ca. tinctorius* in June or early July [19]. This period corresponds to the Australian winter, a period where eggs could potentially be laid on plants. Tsialtas, Michelsen, and Koveos [18] suggested that early infestation is more detrimental to the plants, potentially leading to death. In our no-choice tests, we observed that infested seedlings died, but no complete larval development was possible. Seed destruction was also observed in our no-choice tests on *Ca. tinctorius* in the “flower bud” treatment. In Australia, the oviposition on *Ca. tinctorius* at this growing stage seems unlikely; flowering starts in late spring [19]. However, the larvae found in Greece in June suggest that the females can continue to oviposit during a longer period and/or the newly emerging flies can mate and oviposit on other host plant species. This finding highlights the need for further research on the oviposition period and life cycle of *B. turcica*, particularly in Greece. Further studies are needed to better understand the conditions under which *B. turcica* targets *Ca. tinctorius* in natural settings. Conducting these experiments in both France and Greece, using various *Ca. tinctorius* varieties and planting them at different times, could be crucial for accurately assessing the phenological compatibility or incompatibility with the fly. This approach might also help to explain the outbreak observed by Tsialtas et al. (2013) [18] who reported that up to 30–40% of plants were infested. Unfortunately, our efforts to conduct field exposure experiments with *Ca. tinctorius* were disrupted twice due to human interference with the field experiment. The project’s termination prevented us from repeating these experiments.

Our results contrast with the preliminary assessment of *B. turcica* host range [16]. The method we used for transferring larvae onto the plants differed slightly and may explain the observed differences. We sprayed the plants with water before and after inoculation, creating a more suitable environment for the larvae, which typically develop in the moist, high-humidity rosette crown. This may also explain why the flowering stage of *Ca. tinctorius* allowed larval development; stem ends in a globular flower capitulum enclosed by clasping bracts [36] that can retain humidity. Also, we observed that the larva was also free to move on the plant, contrary to Vitou, Briese, Sheppard, and Thomann [16], where the plants with larvae were sandwiched between two pieces of filter paper to prevent larvae from dropping off the plant. We also tested more growing stages of *Ca. tinctorius* than in the previous study.

Our laboratory tests and field surveys showed that *Ce. solstitialis* is well within the host range of *B. turcica*. The no-choice test showed no difference in larval survival between *Ce. solstitialis* and *Ca. tinctorius* with flower buds, and the choice experiment showed a strong preference for oviposition on *Ce. solstitialis* relative to *Ca. lanatus.* Field surveys undertaken in France and Greece showed that *B. turcica* can naturally infest both *Ce. solstitialis* and *Ce. diffusa* as well as *Ca. lanatus*. However, at Viols-en-Laval, a site where *Ca. lanatus* and *Ce. solstitialis* co-occurred, we detected a higher infestation rate on *Ca. lanatus,* which may indicate a field preference for *Ca. lanatus* over *Ce. solstitialis*.

In our field study, we only considered the infestation rate on each plant species, and so we did not have an estimate of the impact of the fly on *Ce. solstitialis*. The characteristics of the infestation on *Ca. lanatus* and *Ce. solstitialis* in the field seem to be similar as follows: one egg or larva per plant that burrowed either directly into the meristem or along a leaf midrib towards the meristem, depending on where eggs were laid. We can thus hypothesize that the larval development of *B. turcica* may affect the growth and seed production of *Ce. solstitialis* in the same manner as *Ca. lanatus* (i.e., plant mortality, seed production, and reduction in the plant’s relative growth rate [15,17]). *Centaurea solstitialis* is a non-native weed in Australia, considered an invasive and troublesome species in all states [37,38]. Likewise, all *Centaurea* species present in Australia are also non-native species [28]. Consequently, the risk of direct non-target effects on the *Centaurea* species could thus be considered beneficial.

Sheppard and Vitou [17] found no evidence that *Ca. lanatus* rosettes were selected on the basis of plant size and stated that choice for oviposition by *B. turcica* females was not dependent on plant size. In our field survey, we found that females prefer larger rosettes for egg-laying. Plant parameters and/or the developmental stage may play a role in the oviposition of *B. turcica* females on *Ca. tinctorius*. As already observed by Vitou, Briese, Sheppard, and Thomann [16], we found that *B. turcica* could not complete larval development on *Ca. tinctorius* seedlings. Vitou, Briese, Sheppard, and Thomann [16] suggested that *Ca. tinctorius* does not form a true rosette and therefore has insufficient meristem tissue for complete larval development. Based on the ‘mother-knows-best’ hypothesis, which states that female insects choose to oviposit on a host plant that increases the performance of their offspring [39], *B. turcica* females may avoid laying their eggs on *Ca. tinctorius* seedlings in the field. Tsialtas, Michelsen, and Koveos [18] mentioned that “plants infested by *B. turcica* early in their growth may die” without reporting the size of the plants on which oviposition was observed or the frequency of this pattern. A better characterization of the oviposition behaviour of *B. turcica* in the field on *Ca. tinctorius* could be a crucial step to further characterize the risk to this crop. In the no-choice tests, *B. turcica* failed to develop on the closest Australian native members of Cardueae thistle genera, *Rhaponticum australe,* suggesting that this species is not at risk of non-target attack. Like *Ca. tinctorius*, *R. australe* does not form a true rosette. The absence of a true rosette stage could explain the failure of larval development.

The *COI* analysis we conducted was essential in revealing that, in Greece, two closely related species are co-occurring and sharing the same host plant species (*Centaurea* species). This method was particularly useful for accurately identifying specimens collected as eggs or larvae. To our knowledge, this is the first record of *B. brunneilinea* in Greece and the southernmost observation of this species in its native range; this species occurs primarily in the northern parts of the West Palaearctic subregion [40]. However, the taxonomy of *B. brunneilinea* remains unresolved, and this taxon could represent a species complex [41]. Our study suggests that the Greek specimens belong to the same species as the two individuals of *B. brunneilinea* from which *COI* sequences have been lodged in publicly available databases; these individuals were collected in northern Europe, in Finland and Germany, respectively.

## 5. Conclusions

Despite a limited host range, the rosette crown-feeding fly, *Botanophila turcica,* does not fully meet one of the most important criteria of a BCA, safety. We showed that *B. turcica* has a wider host range than previously reported. The fly may have the potential to control both the target weed, *Ca. lanatus,* and *Ce. Solstitialis,* but laboratory tests also indicate that the closely related crop, safflower (*Ca. tinctorius*), is within the insect’s fundamental host range. Based on the current findings, while the risk to *Ca. tinctorius* may be low, it may not be negligible or zero. Further investigations to assess under what conditions *B. turcica* attacks *Ca. tinctorius* may help clarify the level of risk to Australian *Ca. tinctorius* growers. Pending these studies, the final decision on whether or not a BCA is suitable for release in any jurisdiction will rest with the relevant regulators, who must balance the benefits and risks of the release of any biological control agent.

## Figures and Tables

**Table 1:** Sampling details for the *Botanophila turcica* samples used in the molecular characterization.

Sample ID	Host-Plant	Country	Location	GPS Coordinates	Collection Date	Sample Stage	GenBank Accession N°
ClBtFrMu	*Carthamus lanatus*	France	Murles (Herault)	43°40′57.0″ N 3°44′47.0″ E	02/05/1995	Adult	PP830091
CsBtFr1-2	*Centaurea solstitialis*	France	Viols-en-Laval (Herault)	43°45′06.3″ N 3°43′15.4″ E	26/01/2021	Larva	OR226758
ClBtFr1-5	*Carthamus lanatus*	France	Viols-en-Laval (Herault)	43°45′06.3″ N 3°43′15.4″ E	12/01/2021	Larva	=CsBtFr1-2
ClBtFr1-23	*Carthamus lanatus*	France	Viols-en-Laval (Herault)	43°45′06.3″ N 3°43′15.4″ E	15/12/2021	Larva	PP830090
ClBtFr1-28	*Carthamus lanatus*	France	Viols-en-Laval (Herault)	43°45′06.3″ N 3°43′15.4″ E	01/12/2021	Larva	=CsBtFr1-2
ClBtFr2-6	*Carthamus lanatus*	France	Saint-Clément-de-Rivière (Herault)	43°41′26.6″ N 3°50′47.1″ E	19/01/2021	Larva	=CsBtFr1-2
ClBtFr-13	*Carthamus lanatus*	France	Saint-Clément-de-Rivière (Herault)	43°41′26.6″ N 3°50′47.1″ E	16/12/2020	♂ Adult	=CsBtFr1-2
ClBtFr3-8	*Carthamus lanatus*	France	Le Triadou (Herault)	43°44′49.8″ N 3°51′30.3″ E	17/11/2021	Larva	OR226757
CtBtFr2-9	Carthamus tinctorius *	France	Saint-Clément-de-Rivière (Herault)	43°41′26.6″ N 3°50′47.1″ E	16/12/2020	Adult	=CsBtFr1-2
CsBtFr2-11	*Centaurea solstitialis*	France	Saint-Clément-de-Rivière (Herault)	43°41′26.6″ N 3°50′47.1″ E	16/12/2020	♂ Adult	=CsBtFr1-2
CsBtFr3-21	*Centaurea solstitialis*	France	Le Triadou (Herault)	43°44′49.8″ N 3°51′30.3″ E	01/12/2021	Larva	=CsBtFr1-2
CsBtFr3-24	*Centaurea solstitialis*	France	Le Triadou (Herault)	43°44′49.8″ N 3°51′30.3″ E	15/12/2021	Larva	=CsBtFr1-2
CsBtFr1-25	*Centaurea solstitialis*	France	Viols-en-Laval (Herault)	43°45′06.3″ N 3°43′15.4″ E	01/12/2021	Larva	=CsBtFr1-2
CsBtFr1-27	*Centaurea solstitialis*	France	Viols-en-Laval (Herault)	43°45′06.3″ N 3°43′15.4″ E	15/12/2021	Larva	=CsBtFr1-2
DIPYST1	*Centaurea solstitialis*	Greece	Galani, Kozani (Macedonia)	40°22′4.60″ N 21°52′20.84″ E	05/12/2018	Larva	MW590595
DIPYST2	*Centaurea solstitialis*	Greece	Galani, Kozani (Macedonia)	40°22′4.60″ N 21°52′20.84″ E	05/12/2018	Larva	=DIPYST1
DIPYST3	*Centaurea solstitialis*	Greece	Galani, Kozani (Macedonia)	40°22′4.60″ N 21°52′20.84″ E	05/12/2018	Larva	MW590596
DIPYST4	*Centaurea solstitialis*	Greece	Galani, Kozani (Macedonia)	40°22′4.60″ N 21°52′20.84″ E	05/12/2018	Larva	=DIPYST1
DIPYST5	*Centaurea solstitialis*	Greece	Galani, Kozani (Macedonia)	40°22′4.60″ N 21°52′20.84″ E	05/12/2018	Larva	MW590601
DIPYST6	*Centaurea solstitialis*	Greece	Galani, Kozani (Macedonia)	40°22′4.60″ N 21°52′20.84″ E	05/12/2018	Larva	=DIPYST5
DIPYST7	*Centaurea solstitialis*	Greece	Galani, Kozani (Macedonia)	40°22′4.60″ N 21°52′20.84″ E	05/12/2018	Egg batch	MW590602
DIPYST8	*Centaurea solstitialis*	Greece	Galani, Kozani (Macedonia)	40°22′4.60″ N 21°52′20.84″ E	05/12/2018	Egg batch	=DIPYST7
LBOT1	*Centaurea diffusa*	Greece	Kilkis near Thessaloniki (Macedonia)	40°56′20.23″ N 22°50′22.47″ E	19/06/2002	Larva	MW590597
LBOT2	*Centaurea diffusa*	Greece	Kilkis near Thessaloniki (Macedonia)	40°56′20.23″ N 22°50′22.47″ E	19/06/2002	Larva	MW590598
LBOT3	*Centaurea diffusa*	Greece	Kilkis near Thessaloniki (Macedonia)	40°56′20.23″ N 22°50′22.47″ E	19/06/2002	Larva	MW590603
LBOT4	*Centaurea diffusa*	Greece	Kilkis near Thessaloniki (Macedonia)	40°56′20.23″ N 22°50′22.47″ E	19/06/2002	Larva	MW590599
LBOT5	*Centaurea diffusa*	Greece	Kilkis near Thessaloniki (Macedonia)	40°56′20.23″ N 22°50′22.47″ E	20/06/2002	Larva	=LBOT3
LBOT6	*Centaurea diffusa*	Greece	Kilkis near Thessaloniki (Macedonia)	40°56′20.23″ N 22°50′22.47″ E	20/06/2002	Larva	=LBOT2
LBOT7	*Centaurea diffusa*	Greece	Kilkis near Thessaloniki (Macedonia)	40°56′20.23″ N 22°50′22.47″ E	20/06/2002	Larva	MW590600
LBOT8	*Centaurea diffusa*	Greece	Kilkis near Thessaloniki (Macedonia)	40°56′20.23″ N 22°50′22.47″ E	20/06/2002	Larva	=LBOT3
LBOT9	*Centaurea diffusa*	Greece	Kilkis near Thessaloniki (Macedonia)	40°56′20.23″ N 22°50′22.47″ E	20/06/2002	Larva	=LBOT7
LBOT10	*Centaurea diffusa*	Greece	Kilkis near Thessaloniki (Macedonia)	40°56′20.23″ N 22°50′22.47″ E	20/06/2002	Larva	=LBOT2
LBOT11	*Centaurea diffusa*	Greece	Kilkis near Thessaloniki (Macedonia)	40°56′20.23″ N 22°50′22.47″ E	20/06/2002	Larva	=LBOT3

* An individual emerged from host range testing, but the egg was originally collected on *Carthamus lanatus.*

**Table 2:** Results of the field surveys performed in the south of France in two consecutive years (2020–2021 and 2021–2022).

Sites	Plant Species	Year	*n*	Infestation Rate (%)
Viols-en-Laval	*Carthamus lanatus*	2020–2021	270	7.41
		2021–2022	120	5.56
	*Centaurea solstitialis*	2020–2021	270	2.59
		2021–2022	120	5.56
Saint-Clément-de-Rivière	*Carthamus lanatus*	2020–2021	270	27.3
		2021–2022	120	12.5
Le Triadou	*Centaurea solstitialis*	2021–2022	120	16.7

**Table 3:** Results of the no-choice tests for *Botanophila turcica* larvae.

Sub-Tribe	Species—Treatment	Damaged Plants (%)	Type of Damage	Successful Adult Emergence
*Centaureinae*	*Carthamus lanatus*	70 a	Damaged root crown	Yes
	*Carthamus tinctorius*—seedlings	30 ab	Plant death	No
	*Carthamus tinctorius*—leaves	0 b	None	/
	*Carthamus tinctorius*—flower buds	85 a	Destroyed seeds	Yes
	*Centaurea solstitialis*	60 a	Damaged root crown	Yes
	*Rhaponticum* *australe*	42 ab	Damaged stem	No
*Carduinae*	*Onopordum illyricum*	0 b	None	/

The percentages of damaged plants among the host plant species were compared using pairwise Fisher’s exact tests with the FDR multiple testing correction. Different letters within the column indicate a significant difference (*p* < 0.05).

## Data Availability

The data presented in this study are available on request from the corresponding author.

## Supplementary Materials

The following supporting information can be downloaded at: https://www.mdpi.com/article/10.3390/insects16040357/s1, Section S1: Additional details on molecular characterization; Figure S1: Boxplot of minimum interspecific K2P distances over 20 *Botanophila* species and of maximum intraspecific K2P distances from a total of 1530 *COI* sequences retrieved from BOLD; Figure S2: Selected part of the barplot showing the false positive (black) and false negative (green) rate of identification of *Botanophila* species as pre-set thresholds change; Figure S3: Phenological timelines of the rosette sizes (rosette diameter and collar diameter) of both *Ca. lanatus* and *Ce. solstitialis* collected in Viols-en-Laval during our surveys, along with the agent abundance (proportion of plants bearing eggs and larvae). References [23,25,26,27,42,43,44] are cited in the Supplementary Materials.
