# Supplementary material for: Fly in the Ointment: Host-Specificity Challenges for Botanophila turcica, a Candidate Agent for the Biological Control of Saffron Thistle in Australia
Source: Insects. 2025 Mar 28;16(4):357. doi: 10.3390/insects16040357 (PMC12028163; doi:10.3390/insects16040357)
Supplement: Supplementary file 1 [file insects-16-00357-s001.zip › insects-3484848-supplementary.pdf]

# Fly in the Ointment: Host-Specificity Challenges for *Botanophila turcica,* a Candidate Agent for the Biological Control of Saffron Thistle in Australia

**Vincent Lesieur [1,*], Thierry Thomann [1], Mireille Jourdan [1], Javid Kashefi [2] and Marie-Claude Bon [2]**

1 CSIRO European Laboratory, 830 Avenue du Campus Agropolis, 34980 Montferrier sur Lez, France
2 European Biological Control Laboratory, USDA-ARS, 810 Avenue du Campus Agropolis, 34980 Montferrier sur Lez, France
* Correspondence: vincent.lesieur.pro@gmail.com or vincent.lesieur@csiro.au

## Supplementary Material, Section S1

### Materials and Methods

#### *Molecular characterization*

To provide an estimate of the distance threshold, the *Botanophila COI* data were downloaded from BOLD before 23 January 2024 and first filtered by the following criteria: (i) sequences that had not been identified to the species level were deleted; (ii) sequences with names that included the keywords, such as "aff", "cf", "nr", and "spp", were eliminated; (iii) sequences without the label "*COI*-5P" were deleted; (iv) species with numbers of sequences less than three were excluded. Furthermore, the sequences were aligned using MUSCLE as implemented in Mega X (Kumar et al., 2018) and translated to amino acid sequences. Sequences with stop codons, were subsequently deleted. We then calculated the Kimura 2-parameter (K2P) intraspecies mean distances (Kimura, 1980) of the retained species and finally, we discarded species with a maximum intraspecific genetic mean distance greater than the 95th-quantile percentile value. Following the data filtering, a total of 1,530 *COI* data of 521 bp and over 20 *Botanophila* species were obtained. The R package *spider* version 1.4-2 (Brown et al., 2012) and the 'dist.dna' function of the *ape* package (Paradis et al., 2004) was used to estimate pairwise K2P distances for all the sequences in the dataset. Using the base R function 'boxplot()', the intra- and interspecific distances were represented in a boxplot that allows the visualisation of the Barcode gap and the identification of *Botanophila* species based on this gap. Then, to set the

threshold value for the *Botanophila* species that could serve as a reference for threshold-based species delimitation studies, we used the function ‘threshold optimisation’ implemented in *spider* which determines the number of true positives, true negatives, false negatives, false positives and the cumulative error (false negatives + false positives) identification for a given threshold.

To estimate the number of species in the final dataset and to explore molecular species delimitation, three different methods were used. First, we used Automatic Barcode Gap Discovery (ABGD) developed by Puillandre et al. (2012). This tool uses pairwise distance to detect a barcode gap, this barcode gap is used as a threshold to separate the sequences into putative species (i.e. genetically homogeneous groups). The procedure is recursively applied to the previously obtained groups until no more partitioning can be done. The analysis was conducted through uploading a sequence alignment on the ABGD web server at https://bioinfo.mnhn.fr/abi/public/abgd/abgdweb.html). We computed a matrix of pairwise distances using the K2P model (Kimura, 1980) and ABGD was run with the default settings (Pmin = 0.001, Pmax = 0.1, Steps = 10, X (relative gap width) = 1.5, Nb bins = 20). The recently developed Assemble Species by Automatic Partitioning (ASAP) is a distance-based method that uses threshold values to distinguish between interspecific divergence and intraspecific variation (Puillandre et al., 2021). The method indicates the presence of a barcode gap and clusters the samples into putative MOTUs within partitions. In addition, partitions are ranked according to an ad-hoc score calculated from the probabilities of groups to be panmictic species and the widths of barcode gaps (Puillandre et al., 2021). As input for ASAP, our sequence alignment was used with two additional sequences added for each outgroup (*B. biciliaris*, GenBank accession numbers: MZ611336 and MG673719; *B. fugax*, GenBank accession numbers: MG673853 and MZ625519 and *B. helviana*, BOLD identifiers: CBG-A11493-D03 and CHARS00385-B11). The analysis was run with default parameters default parameters, based on K2P distances (available at https://bioinfo.mnhn.fr/abi/public/asap/asapweb.html). The Bayesian implementation of the Poisson tree processes (bPTP) model for species delimitation (Zhang et al., 2013) was also used. This model uses coalescence theory and estimates the speciation rate directly from the number of substitutions. It assumes that each substitution has a small probability of generating a speciation event. Consequently, the number of substitutions between species is expected to be significantly higher than within species. The ML phylogeny (without haplotype duplicates) obtained with PhyML was used as input. The analysis was run on a web server for PTP (available at http://species.h-its.org/ptp/) with 300,000 MCMC generations, a thinning value of 100 and a

burn-in of 25%. As recommended by the developers, the convergence of the MCMC chain was confirmed visually.

## Results

### *Molecular characterization*

Intraspecific K2P distances was found generally lower than the interspecific K2P distances (ranging from 0.0000 to 0.0446 (median = 0.0021) *vs*. 0.0000 to 0.1220 (median = 0.0470) indicating a barcode gap in the dataset and making the *CO1* barcode region adequate to delimit *Botanophila* species in our study. The boxplot of the maximum intraspecific K2P distances and the minimum interspecific distances of congeneric species is shown in Figure S1.

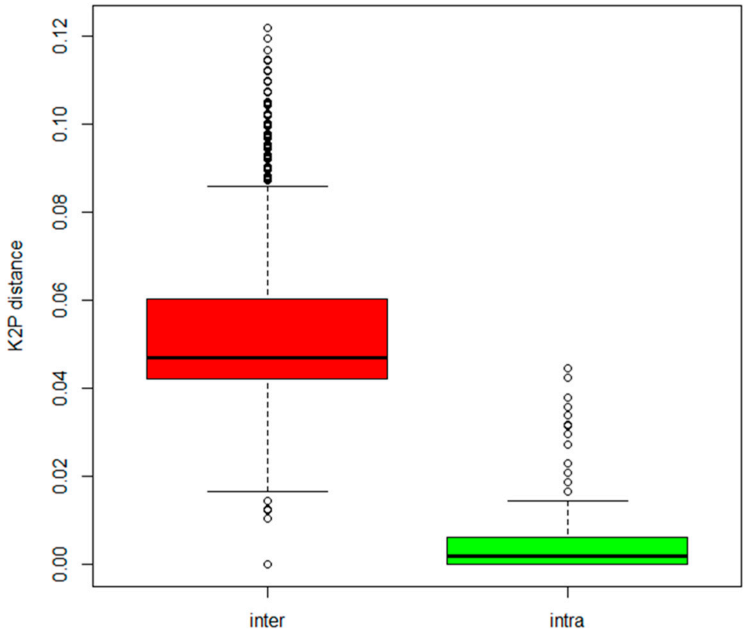


Figure S1. Boxplot of minimum interspecific K2P distances over 20 *Botanophila* species and of maximum intraspecific K2P distances from a total of 1,530 *COI* sequences retrieved from BOLD (https://www.boldsystems.org/).

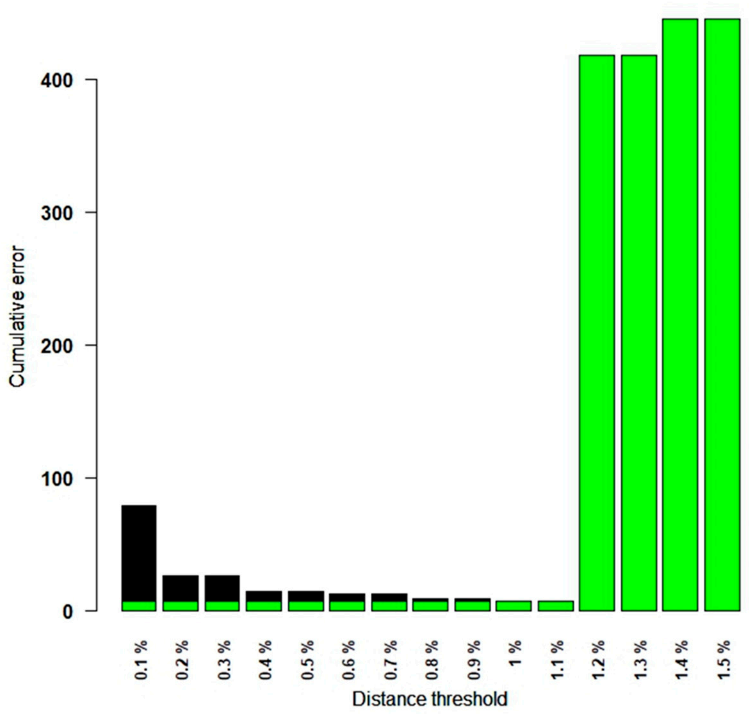


Figure S2: Selected part of the barplot showing the false positive (black) and false negative (green) rate of identification of *Botanophila* species as pre-set thresholds change.

The optimum threshold for our *Botanophila* dataset is between 1% and 1.1% K2P distance (Fig. S2). These thresholds have the lowest cumulative error at 7 false negative identifications (i.e. non-conspecific species within threshold distance of query) out of 1,530 sequences and may represent thresholds to delimit most *Botanophila* species.

### ***Field surveys***

We did not observe any significant differences between *Ca. lanatus* and *Ce. solstitialis* in terms of the seasonal dynamics of the fly (Supplementary Material, Figure S2). However, at Viols en Laval, *B. turcica* was detected on *Ca. lanatus* two weeks earlier than on *Ce. solstitialis*.

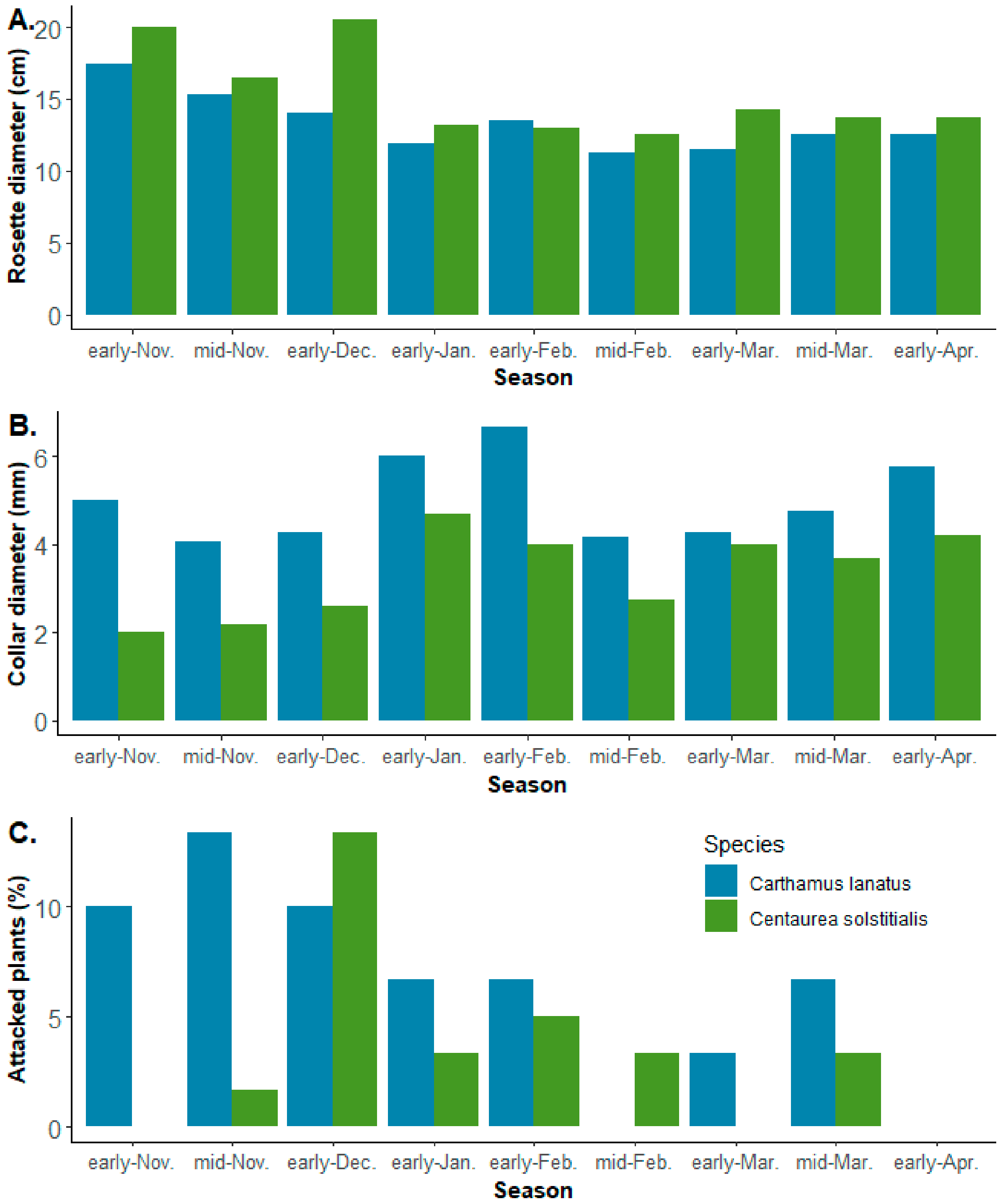


Figure S3. Phenological timelines of the rosette sizes (rosette diameter and collar diameter) of both *Ca. lanatus* and *Ce. solstitialis* collected in Viols-en-Laval during our surveys, along with the agent abundance (proportion of plant bearing eggs and larvae).

**References**


Brown, S.D., Collins, R.A., Boyer, S., Lefort, M.C., Malumbres-Olarte, J., Vink, C.J., Cruickshank, R.H., 2012. Spider: an R package for the analysis of species identity and evolution, with particular reference to DNA barcoding. Molecular Ecology Resources 12, 562-565.

Kimura, M., 1980. A simple method for estimating evolutionary rates of base substitutions through comparative studies of nucleotide sequences. Journal of molecular evolution 16, 111-120.

Kumar, S., Stecher, G., Li, M., Knyaz, C., Tamura, K., 2018. MEGA X: molecular evolutionary genetics analysis across computing platforms. Molecular biology and evolution 35, 1547-1549.

Paradis, E., Claude, J., Strimmer, K., 2004. APE: analyses of phylogenetics and evolution in R language. Bioinformatics 20, 289-290.

Puillandre, N., Brouillet, S., Achaz, G., 2021. ASAP: assemble species by automatic partitioning. Molecular Ecology Resources 21, 609-620.

Puillandre, N., Lambert, A., Brouillet, S., Achaz, G., 2012. ABGD, Automatic Barcode Gap Discovery for primary species delimitation. Molecular ecology 21, 1864-1877.

Zhang, J., Kapli, P., Pavlidis, P., Stamatakis, A., 2013. A general species delimitation method with applications to phylogenetic placements. Bioinformatics 29, 2869-2876.
